# Storm deposition layer on the Fujian coast generated by Typhoon Saola (2012)

**DOI:** 10.1038/srep14904

**Published:** 2015-10-08

**Authors:** Yunhai Li, Haidong Li, Lei Qiao, Yonghang Xu, Xijie Yin, Jianhua He

**Affiliations:** 1 Open Laboratory for Coast & Ocean Environmental Geology, Third Institute of Oceanography, State Oceanic Administration, Xiamen, China; 2 College of Earth Sciences, Jilin University, Changchun, China; 3 Department of Natural Resource Ecology & Management, Oklahoma State University, Stillwater, OK, USA; 4 Laboratory of Marine Isotopic Technology and Environmental Risk Assessment, Third Institute of Oceanography, State Oceanic Administration, Xiamen, China

## Abstract

Typhoons have a significant effect on the marine depositional environment and depositional process. In this paper, we used the high-resolution Chirp sonar sub-bottom profiler and radioisotope detection techniques to examine the storm-deposited layer formed in the seawater near the path of Typhoon Saola along the coast of Fujian, China. The thickness of the typhoon-deposited layer acquired using these two methods was 10–25 cm. The thickness, sediment grain size, and δ^13^C values of the deposited sedimentary layer indicated that it was mainly matter from the re-suspension and redistribution of seafloor sediments. The particle sizes of the sediments in the storm-deposited layer became coarser, indicating that the fine-grade compositions spread over a wider range out of the coastal zone.

Typhoons have a large effect on coastal sediment budgets and the characteristics of strata preserved in the geological record^1^. During a typhoon, strong cyclonic wind stress accelerates sediment re-suspension, and heavy precipitation accelerates terrigenous particle supply through run-off in coastal areas. The concentration of suspended particles in the seawater column might increase by several decades to a hundred times during a typhoon, and the large suspended particles might be re-transported over a wide region by typhoon-induced currents and re-deposited to form a storm-event sedimentary sequence^2^,^3^,^4^,^5^,^6^,^7^,^8^,^9^. The geological characteristics of the sediments in a storm-event sedimentary sequence are different from normal sediments^10^,^11^,^12^. Among the differences, radioactive isotopes (such as ^210^Pb, ^137^Cs, ^7^Be and ^234^Th), which can accurately identify newly deposited storm-event layers^10^,^11^,^12^,^13^, are effective indices to discriminate storm-event sedimentary sequences. High-resolution marine geophysical technologies (such as a Multiple-Beam System and Chirp Sonar Sub-bottom Profiler System) are also used to detect the effects of typhoon events on seafloor topography and coastal landforms^1^,^7^,^8^. According to long-term meteorological records, an average of 4 to 5 typhoons affect the Fujian coast, China annually^14^, which significantly impacts the modern sedimentary system, including sediment transport and deposition processes. The Fujian coast acts as a natural laboratory for studying the modern typhoon-induced storm-event sedimentary processes because of its thick mud sediments and well-preserved sedimentary records. However, because of the insufficient ability to forecast typhoons and shipboard operation difficulties under severe weather conditions, there are no studies regarding typhoon-event storm deposition layers on the Fujian coast.

The present study comprehensively utilized a high-resolution Chirp Sonar Sub-bottom Profiler System and ^210^Pb radioactive isotopes to identify the storm sedimentary sequence generated by Typhoon Saola along the Fujian coast in 2012. The source of sediments in the storm layer and the deposition process during the typhoon are discussed by analyzing the distributions of sedimentary thickness, sediment grain size and δ^13^C in the storm deposition layer. The results may contribute to a better understanding of the formation and identification of storm deposition layers in coastal areas.

## Typhoon Saola

**Figure 1:**
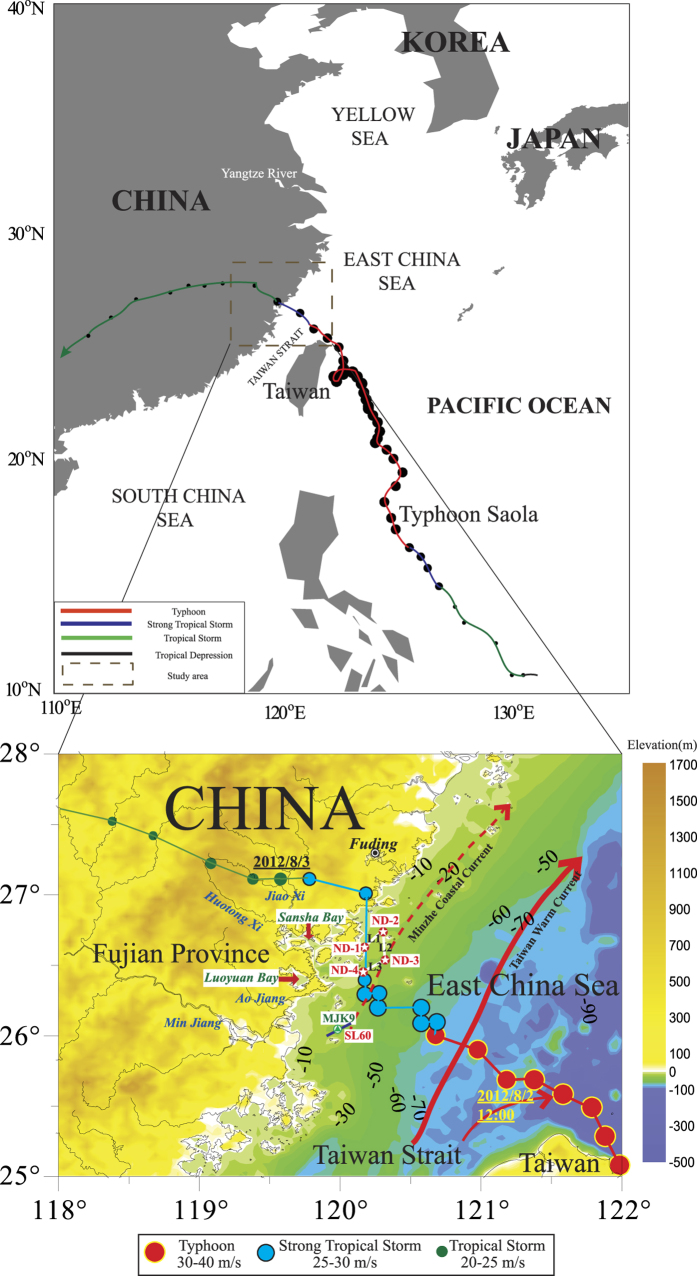
The path of Typhoon Saola in 2012 (modified from the typhoon path data at http://map.weather.gov.cn/). The red solid line represents the Taiwan Warm Current. The red dashed line represents the Minzhe Coastal Current in summer. The white solid lines represent the L1, L2 and L3 seismic lines and the blue solid line represents the SL60 seismic line. The red dots represent the 4 box-type sampling stations (ND-1, ND-2, ND-3 and ND-4), and the green dot represents the core MJK9. The main rivers in the study area include the Min River, Ao River, Huotong Xi and Jiao Xi. Sansha Bay and Luoyuan Bay are semi-enclosed bays with a maximum water depth of more than 60 m. This figure was drawn using Surfer (version 11.6).

Typhoon Saola was first formed east of the Philippines on July 28, 2012 and moved northwest after its formation. It was strengthened to a typhoon on the afternoon of July 30 and intensified to a strong typhoon at 14:00 on August 1. Typhoon Saola landed on Hualien, Taiwan at 19:00 on August 1. The typhoon gradually weakened and landed second time on Fuding, Fujian at 22:00 on August 2. The maximum wind at the center of the typhoon at the second landing was 33 m/s. The typhoon continued to move into inland Fujian after landing and weakened into a tropical storm. The numbering stopped on the evening of August 3 (Fig. 1)^15^,^16^. Typhoon Saola had a high intensity but with a slow moving speed. In combination with a southwest monsoon, the local rain was heavy, affecting a wider spatial range over a long time period. The typhoon significantly affected the coastal marine sedimentary environment by altering material supply, transportation and deposition.

## Results

The study area was located in the mud depo-area off the Zhejiang-Fujian coast (Fig. 1), where the seafloor sediments were dominated by fine-grained clayey silt and silty clay. The thickness of muddy sediment, which was mainly discharged by Yangtze River and transported southward by the coastal current (namely the Minzhe Coastal Current), was more than 30 m in the deposition center and the sedimentary structure of stratum of the upper sediment layer was relatively uniform without any sub-reflection^17^,^18^.

**Figure 2:**
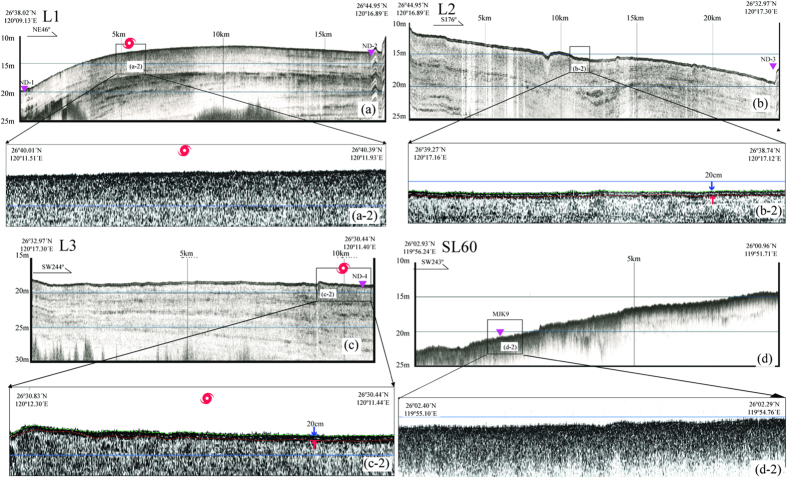
L1 (a), L2 (b), L3 (c) and SL60 (d) seismic profiles. The section location and extension direction are shown in Fig. 1. The serrated undulation on the surface of the seafloor is the reflection of the surface waves and does not represent real submarine undulations. The red typhoon symbol represents the location of Typhoon Saola. The inverted pink symbol represents the location of the 5 box-type sampling stations. The vertical axis represents the distance from sea level, the longitude and latitude on the two ends represent the beginning position of the measuring line, and the -2 figure is the zoomed-in view of the cross section. Note that there is a double-reflection phenomenon in sections L2 and L3 but not in L1 and SL60, and the reflection layer is approximately 20 cm thick.

The characteristics of the shallow stratum profile on the 4 survey profilers are shown in Fig. 2. Typhoon Saola passed through profilers L1 and L3, and its path was generally parallel to profiler L2 (Fig. 1) (measured on August 14, 2012). And the profiler SL60 (measured on August 19, 2012), located in the inner-shelf off the Minjiang Estuary, was treated as a reference line (far away from the typhoon path and beyond the typhoon’s direct impact). Profiler L1 (18.59 km), located in the coastal shallow water, had a water depth between 12 and 20 m from the west side to east side. The physical characteristics of sediment and the structure of stratum were relatively uniform in the whole upper-most 10–20 m layer of the chirp reflection images (Fig. 2a; a-2). However, in the profilers L2 (22.29 km) and L3 (10.91 km), extending from the coastal shallow water to deep-water area from the north side to the south side, there exist double reflections on the near-surface with the thickness of upper sediment layer was about 20 cm in the chirp reflection images (Fig. 2b; b-2 and 2c; c-2). The double reflections both in profilers L2 and L3 were distributed across the entire profilers and slightly weakened near the coast (Fig. 2b; b-2 and Fig. 2c; c-2). In the reference profiler SL60 (8.38 km), which extended from the coastal shallow water to deep-water area, the physical characteristics of sediment and the structure of stratum were relatively uniform in the whole upper-most 10–20 m layer in agreement to the profiler L1 of the chirp reflection images (Fig. 2d; d-2).

Due to the uniform physical characteristics of seafloor sediments in the study area, the reflection of stratum should be uniform without any sub-reflections just as shown in the reference profiler SL60 (Fig. 2d; d-2) and other previous studies^17^,^18^. On the contrary, in the Typhoon Saola active area (Profilers L2 and L3), a distinct double reflections appeared in the near-surface sediments in the relatively deep-water area (Fig. 2b; b-2 and Fig. 2c; c-2). The double reflections indicate significant changes in the physical properties of the sediments, which might be the newly formed or transformed sediment layer caused by the typhoon. The thickness of the newly sediment layer could be discerned in the shallow stratum profiles and was relatively thin (approximately 20 cm in thickness). However, there were no double reflections in the profiler L1, which is also located in the typhoon-impacted area. Possible mechanism will be analyzed in the discussion section by elaborating and comparing the distributions of sediment physical characteristics in different cores (Fig. 2a; a-2).

**Figure 3:**
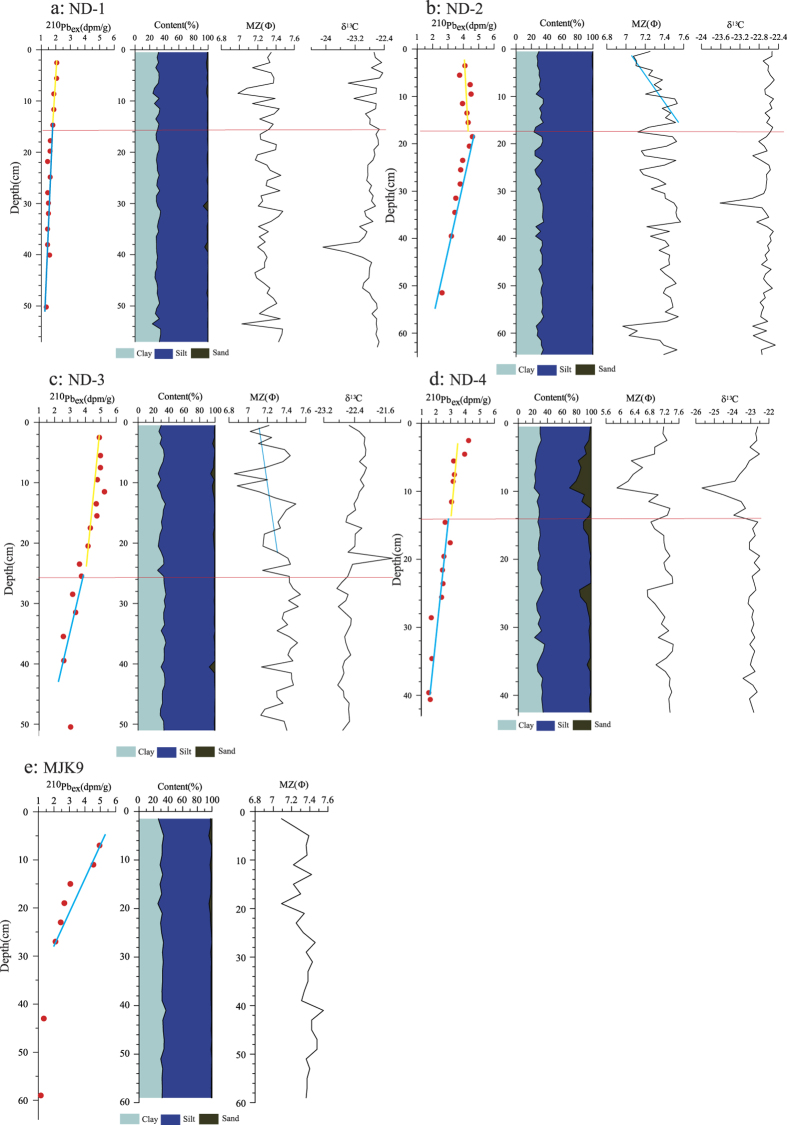
The down-core variations of ^210^Pb_ex_, grain size, average particle size and δ^13^C in the cores of ND-1 (a), ND-2 (b), ND-3 (c), ND-4 (d) and MJK9 (e).

The distribution features of ^210^Pb_ex_ activity, particle size and δ^13^C in the 4 box-type samples collected in the study area, along with a reference core (MJK9, collected in the profile SL60 in April, 2010) are shown in Fig. 3. The sediments were relatively homogeneous and mainly composed of fine-grained clayey silt in all the 5 cores, consistent with previous findings^17^,^18^. The distributions of δ^13^C is shown as a steady pattern with little fluctuations from the top to bottom of sediments with value generally ranging from −23 to −22.4‰ in the 4 cores (Fig. 3). In the core ND-4, the value of δ^13^C was decreased to around -26‰ with the increase of sand content in the 2–14 cm depth of sediments (Fig. 3d). The distributions of ^210^Pb_ex_ (less than 2 dpm/g) and average grain size (range from 7 to 7.4 Φ) in the core ND-1 were relatively uniform from the top to bottom (Fig. 3a). In the cores of ND-2, ND-3 and ND-4, the sediments can be divided into two parts according to the distributions of ^210^Pb_ex_ and grain size. The thickness of upper parts was 18, 26 and 14 cm, respectively, in the core of ND-2, ND-3 and ND-4 (Fig. 3a–c). The boundary of the two layers corresponded with color variations (brown in upper layer and dark brown in bottom layer) in the sediments (Fig. 3). The content of ^210^Pb_ex_ was relatively high (about 4, 4–5 and 3–4 dpm/g, respectively, in the core of ND-2, ND-3 and Nd-4) without significant vertical decay in the top sediments, while it was gradually reduced in the bottom layer. In the upper sediment layer, the grain size was significantly larger than that in the lower sediment layer in the 3 cores. The variations of the content of ^210^Pb_ex_, average grain size and sand content in the sediments were similar in the 3 cores. The distribution of ^210^Pb_ex_ in the reference core MJK9 showed a different pattern, with high value and significant vertical decay (from 5 decreased to 2 dpm/g) in the top 28 cm, while it was low and stable (less than 2 dpm/g) in the bottom layer. The average grain size in the upper layer was slightly larger than that in the bottom layer (Fig. 3e).

Based on the distributions of ^210^Pb_ex_ content, color and average grain size, the sediments of the 4 cores in typhoon-active area can be divided into 2 parts. The top part was generally 10–25 cm thick, with the ^210^Pb_ex_ content relatively high and uniform vertically, which showed typical characteristics of mixed sediment layer. Judging from fresh yellow brown color and the high ^210^Pb_ex_ content, this layer might be the product of strong perturbation or simultaneous short-term deposition caused by typhoon process. The sediment grain sizes in the upper layer of ND-2, ND-3 and ND-4 were larger than those in the bottom part, which indicated a coarsening of sediment (Fig. 3b–d). In the bottom part, the ^210^Pb_ex_ content gradually decreased with increasing depth, and the color was mainly dark brown, which indicated that the sediments in this part was generally stable and continuously deposited. There were significantly rough fractures between the two parts (in Fig. 3). In addition, the thickness of the upper sediment layer in the 4 box-type samples corresponded well with the shallow stratum profile, which might be the storm-deposited or storm-transformed sedimentary layer formed by Typhoon Saola. However, the distribution of ^210^Pb_ex_ content and average grain size of sediments in the reference core MJK9 showed a typical stable and continuous deposition signature without sediments mixing and fresh brown color.

## Discussion and Conclusions

The Chirp sonar sub-bottom profiler can distinguish and detect the reflections of shallow stratum due to the different physical properties of sediments. The ^210^Pb_ex_ content in sediments reflects the depositional features. The results from the two methods corresponded well to each other in this study. From the shallow stratum profile in the Typhoon Saola active area and the distribution of ^210^Pb_ex_ content in the sediments, a 20 cm-thick sediments formed (or disturbed) by Typhoon Saola was distinguished in the mud depo-area off the Zhejiang-Fujian coast. The distribution patterns of ^210^Pb_ex_, showing that a gradually radioactive decaying sediment layer covered by a stable and high-content ^210^Pb_ex_ top sediments in the typhoon-affected area, were significantly different from that in a reference station (MJK9), in which the ^210^Pb_ex_ content gradually decayed from top to bottom sediments (Fig. 3). It was puzzling that there were no double reflections in the profiler L1 as mentioned above (Fig. 2a; a-2). The ^210^Pb_ex_ content in the whole core of ND-1 was very low (<2 dpm/g) compared with other cores (mainly higher than 4 dpm/g). This distribution pattern indicated that the sediment in the top of core ND-1 was mainly old deposit, and there was no sediment deposition during the typhoon processes. At the other stations, the variations in ^210^Pb_ex_ content indicated that the storm-deposited (disturbed) layer was relatively thick. The difference in the physical properties between the upper and bottom sediment layers was significant, and the storm-deposited (disturbance) layer was identified on the shallow stratum profile.

The strong dynamic processes of the typhoon disturbed the seafloor sediments and increased the suspended particle content in the seawater. In addition, the heavy rainfall caused by the typhoon increased the sediment flux into the sea and the particle content in the seawater. The suspended particles were carried by typhoon-induced current and spread in a large area. After the typhoon, the suspended particles deposited quickly and formed the storm-deposited layer. The δ^13^C value of the sediments in the 4 cores sediment did not vary significantly (Fig. 3), which might be an indicator that the material source in the entire sample was homologous. According to the previous studies, the seafloor sediments in the study area were mainly from Yangtze River, although there are several small rivers run into the sea around the study area, including the Min River, Ao River, Huotong Xi and Jiao Xi. Among these small rivers, the discharge of water and sediment of the Min River are the largest (average 7.5 Mt per year, while all other rivers yield approximately 3 Mt per year). The sediments from the Min River are mainly deposited near the estuary and transported south. The sediments discharged by the Huotong Xi and Jiao Xi are mainly deposited in Sansha Bay. As the sediments in the study area were mainly from the Yangtze River, carried by southward coastal currents in Fujian and Zhejiang, the mineralogical and geochemical properties of sediments were relatively stable and uniform^18^. Therefore, the sediments in the storm-deposited layer formed by Typhoon Saola were mostly likely the re-suspension and re-distribution of seafloor sediments in typhoon-affected seawater that were deposited previously by Yangtze River. Except for ND-1, the sediment particle size in the top layer at the stations became coarser than the lower layer (Fig. 3), possibly because transporting coarse re-suspended sediments from coastal area to wider seawater was harder than transporting fine particles. Using the detection data of the shallow stratum profile over a large area, we roughly calculated the redistributed amount of sediment caused by Typhoon Saola and the thickness of the storm-deposited layer can be accurately determined through the ^210^Pb_ex_ content. This process provides a new approach for evaluating the impact of typhoon on the modern sedimentary process.

The strong dynamic process during the typhoon period disturbed the seafloor sediment and caused the sediment re-suspension. The re-suspended particles were carried by typhoon-driven currents and spread over a wider area, which increased significantly the transport flux of the sediment and promoted the materials re-distribution in the coastal and continental area, especially in the longitudinally direction to the outer continental shelf, which might be limited by the strong northward-flowing Taiwan Warm Current under the calm marine situation in summer^6^. In the shallow coastal area, due to the strong sediment re-suspension and the re-transport, the newly sediment is hardly to be reserved and form new sedimentary stratum. However, in the relatively deep water area, the re-suspended and re-transported sediments, especially the relatively coarse ones, can deposit quickly and form a storm-induced sediment stratum.

In summary, this study used the Chirp sonar sub-bottom profiler and radioisotope method to determine the storm-deposited layer formed (disturbed) by Typhoon Saola in a mud depo-area off the Zhejiang-Fujian coast. By combining the thickness of the sediment layer (detected by both sonar profiler and ^210^Pb_ex_ content) and the distributions of sediment grain size and the δ^13^C values, we preliminarily examined the material source and deposition process of the storm sediment layer. The results indicated that a 10–25 cm storm-deposited (disturbed) layer was formed near the path of Typhoon Saola in the Fujian coastal area, and the sediment mainly came from the re-suspension of seafloor materials. The grain size in the storm-deposited layer became coarser, which indicated that the fine-grained compositions spread across a wider range after the typhoon, thus increasing the material transport flux in the seawater. Typhoons play a significant role in the modern marine sedimentary process, and the combining use of the shallow stratum profile detection technique and radioactive geochemical method provides a new approach for estimating typhoon effects on modern sedimentary processes.

## Methods

A high-resolution EdgeTech 0512i Chirp Sonar Sub-bottom Profiler (frequency range: 1–10 kHz/5 ms) was used to obtain 3 seismic lines (51.79 km) on August 14, 2012 around the path of Typhoon Saola in a mud depo-area off the Zhejiang-Fujian coast, and a reference seismic line SL60 (8.38 km) was obtained on August 19, 2012 in the inner-shelf off the Minjiang Estuary using the same profiler (Fig. 1). An acoustic velocity of 1500 ms^−1^ was used to calculate water depth and sediment thickness^16^. Five shallow cores (43–64 cm) were collected using a box corer along 4 seismic lines, among them the core MJK9 in the line SL60 was collected on April 25, 2010 and its detail information can be found in a previous study^19^ (Fig. 1). The cores were cut into 1-cm intervals in the laboratory, and the grain size and δ^13^C of every subsample was measured using a laser particle size analyzer (Mastersizer, 2000), with a measuring error within 3%, and an elementary analysis-isotope ratio mass spectrometers (EA-IRMS) (Flash EA 1112 HT-Delta V Advantages), with a measuring error within ±0.2%. The ^210^Pb radioisotope activities of the sediment were analyzed in 3- to 4-cm intervals by gamma spectrometry.

## Additional Information

**How to cite this article**: Li, Y. *et al.* Storm deposition layer on the Fujian coast generated by Typhoon Saola (2012). *Sci. Rep.* **5**, 14904; doi: 10.1038/srep14904 (2015).
